# Efficacy of erector spinae plane block in pain management for patients with herpes zoster: a systematic review and meta-analysis

**DOI:** 10.1016/j.bjane.2025.844598

**Published:** 2025-02-18

**Authors:** Alexandre Yamada Fujimura Júnior, Carolina Braga Moura, Arnaldo Bastos dos Santos

**Affiliations:** a Faculdade de Medicina de Marília (FAMEMA), Departamento de Medicina, Marília, SP, Brazil; b Universidade Federal Fluminense (UFF), Departamento de Neurologia, Niterói, RJ, Brazil; c Hospital Sírio Libanês, Departamento de Anestesiologia, São Paulo, SP, Brazil

**Keywords:** Nerve block, Anesthesia, conduction, Herpes zoster, Pain, Neuralgia, postherpetic

## Abstract

**Objectives:**

Systematic review and meta-analysis to evaluate the efficacy of the Erector Spinae Plane Block (ESPB) in managing pain related to Herpes Zoster.

**Methods:**

We systematically searched PubMed, Embase, Cochrane Library, and CNKI for randomized trials comparing ESPB plus standard clinical treatment with clinical treatment alone. The population included patients with acute infection and those with Postherpetic Neuralgia (PHN). The primary outcome was pain intensity, and secondary outcomes included analgesic consumption. Mean Difference (MD) was used for continuous outcomes, and Risk Ratio (RR) for binary outcomes.

**Results:**

Seven trials with 362 patients were included. ESPB significantly reduced pain up to eight weeks (MD = -1.21; 95% CI -2.17 to -0.24; I^2^ = 89%). In the subgroup analysis of patients in the acute stage, the benefit seemed to extend with pain reduction lasting up to 12-weeks (MD = -1.49; 95% CI -2.61 to -0.37; I^2^ = 0%), and a reduction in the incidence of PHN (RR = 0.49; 95% CI 0.28 to 0.85; I^2^: 0%). In the PHN subgroup, pain reduction was notable only at four weeks (MD = -1.08; 95% CI -1.81 to -0.35; I^2^ = 86%). ESPB also reduced acetaminophen (MD = -0.6 g.day^-1^; 95% CI -1.05 to -0.14; I^2^ = 49%) and pregabalin consumption (-68.58 mg.day^-1^; 95% CI -127.18 to -9.97; I^2^ = 41%) over 12 weeks.

**Conclusion:**

ESPB seems to provide pain relief in Herpes Zoster patients, with a prolonged benefit in the acute stage. Also, ESPB reduced the need for analgesics over 12 weeks. More research is needed to corroborate this practice.

**Study Registration Number and Date:**

This article was prospectively registered in PROSPERO (www.crd.york.ac.uk/prospero, CRD42024566674).

## Introduction

Herpes zoster, caused by the reactivation of the varicella-zoster virus in the sensory ganglia of cranial nerves and dorsal root ganglia, manifests as a painful rash along the affected dermatome. In addition to pain, this condition severely impacts quality of life, particularly in the elderly, who are more susceptible to complications such as Postherpetic Neuralgia (PHN), a chronic neuropathic condition.^1^, ^2^, ^3^, ^4^

Although vaccination has proven effective in reducing the incidence of herpes zoster, vaccine uptake remains low, with only 56% of eligible patients receiving it.^5^ Consequently, interventional therapies targeting different stages of the disease are still essential, especially in refractory cases where inappropriate use of analgesics, including anti-inflammatory drugs and opioids, can result in more harm than benefit.^6^

Anesthetic techniques like paravertebral and epidural blocks have demonstrated efficacy in pain relief and in reducing the incidence of PHN.^7^^,^^8^ However, these techniques require advanced expertise and carry a higher risk of complications.^9^^,^^10^

The Erector Spinae Plane Block (ESPB) has recently emerged as a safer, easier alternative with fewer complications, such as pneumothorax and hematoma.^11^^,^^12^ Given these potential advantages, we conducted a meta-analysis to evaluate the effectiveness of ESPB in managing pain associated with herpes zoster.

## Material and methods

### Protocol and registration

This systematic review and meta-analysis was conducted and reported in accordance with the Cochrane Collaboration Handbook for Systematic Reviews of Interventions and the Preferred Reporting Items for Systematic Reviews and Meta-Analysis (PRISMA) guidelines.^13^ The review aimed to evaluate the efficacy of the ESPB in patients with pain related to Herpes Zoster infection. This study was prospectively registered in PROSPERO with number CRD42024566674.

### Eligibility criteria

Studies were included in this review if they met the following criteria: 1) Randomized Clinical Trials (RCTs); 2) Enrolled patients with pain related to herpes zoster in two contexts: acute herpes zoster (reactivation of a viral infection with severe pain in a specific dermatome associated with vesicular erythema) and PHN (persistent pain lasting more than three months after the resolution of acute infection and dermatological lesions); 3) Compared clinical treatment combined with the application of ESPB to clinical treatment alone; and 4) Assessed at least one of the targeted outcomes of this meta-analysis. Studies without a control group or with overlapping patient populations were excluded. No restrictions were applied regarding the date or language of publication, as part of an effort to broaden the search.

### Search strategy and study selection

We conducted a systematic search of the MEDLINE, Cochrane, Embase, and China National Knowledge Infrastructure (CNKI) databases from their inception until July to September 2024. The search strategy used the following terms: “erector spinae plane block”; “erector spinae block”; “herpes-zoster”; “herpes zoster”; “postherpetic”; “varicella zoster virus”; “Varicella-Zoster Virus” and “Chickenpox”. The complete search strategy, using Boolean operators and specific models adapted for each database searched, is available in Supplementary Table 1.

All identified articles were systematically evaluated according to the inclusion and exclusion criteria. Article selection was performed independently by two authors (A.Y and A.B), with any disagreements resolved by consensus. To enhance the search for studies, the authors reviewed the references and related studies of the included articles to locate additional studies that met the inclusion criteria.

### Data extraction

Two reviewers (A.Y and C.B) independently read the included studies and extracted data on 1) Pain scale over each available week or month; 2) Consumption of any analgesics, whether opioids or anti-inflammatory drugs, or any neuroleptics used by patients during clinical follow-up; 3) Number of patients who developed PHN; 4) Adverse effects, including minor ones such as nausea, vomiting, dizziness, headache, or transient hypotension, and major ones such as bleeding and pneumothorax. Additionally, data were collected regarding the type of anesthetic drug used for ESPB, the number of applications performed during the study, the clinical treatment protocol, and key epidemiological characteristics, including the age and sex of the patients. All extracted data were cross verified for accuracy by a third reviewer (A.B).

Regarding the pain score, we used the Visual Analog Scale (VAS), graded from 0 to 10, as the standard for representing our forest plots. We considered, based on a previous study, the equivalence between VAS and the Numeric Rating Scale (NRS).^14^ For continuous outcomes, we extracted the mean and standard deviation for each group. When only the median and quartiles were available, we converted them into mean and standard deviation.^15^^,^^16^

To calculate the daily mean intake of analgesics used over 12 to 11 weeks of follow-up, we used the weighted mean,^17^ in the same way, that each daily mean reported over different time intervals was adjusted according to the duration of each interval. Thus, longer time intervals had more weight in calculating the average over 12 weeks. In the same way, we calculated the combined average standard deviation over the 12-week period using a more complex equation.^18^

### Quality assessment

Quality assessment of RCTs was performed using the Cochrane Collaboration's tool^19^ for assessing risk of bias in randomized trials, in which studies are scored as high, low, or some concerns of risk of bias in 5 domains: selection, performance, detection, attrition, and reporting biases. This risk of bias evaluation was performed independently by two authors (A.Y and C.B) with disagreements resolved by consensus. To create the risk of bias figure, we used the Robvis tool.^20^

### Data analysis

The effects of ESPB on continuous outcomes were evaluated using the Mean Difference (MD) with a 95% Confidence Interval (95% CI), while binary outcomes were assessed using the Risk Ratio (RR) with a 95% CI.

For binary outcomes, we employed the Mantel-Haenszel method, and for continuous outcomes, we used the inverse-variance method. Heterogeneity was assessed through Cochran's *Q* test, I^2^ statistics, and Tau-squared, utilizing the restricted maximum likelihood estimator. Heterogeneity was categorized as low (I^2^ = 0%–25%), moderate (I^2^ = 26%–50%), or high (I^2^ > 50%). The fixed-effects model was used for outcomes with low heterogeneity (I^2^ < 25%) and the random-effects model for studies with moderate to high heterogeneity (I^2^ > 25%). All statistical analyses were performed using Review Manager version 5.4 (Cochrane Center, The Cochrane Collaboration, Denmark).

We also conducted subgroup analysis and sensitivity analysis to evaluate the effect of ESPB in the different clinical phases of herpes zoster. Due to the limited number of studies for each outcome, we did not perform publication bias analysis.

## Results

### Study selection and characteristics

**Figure 1:**
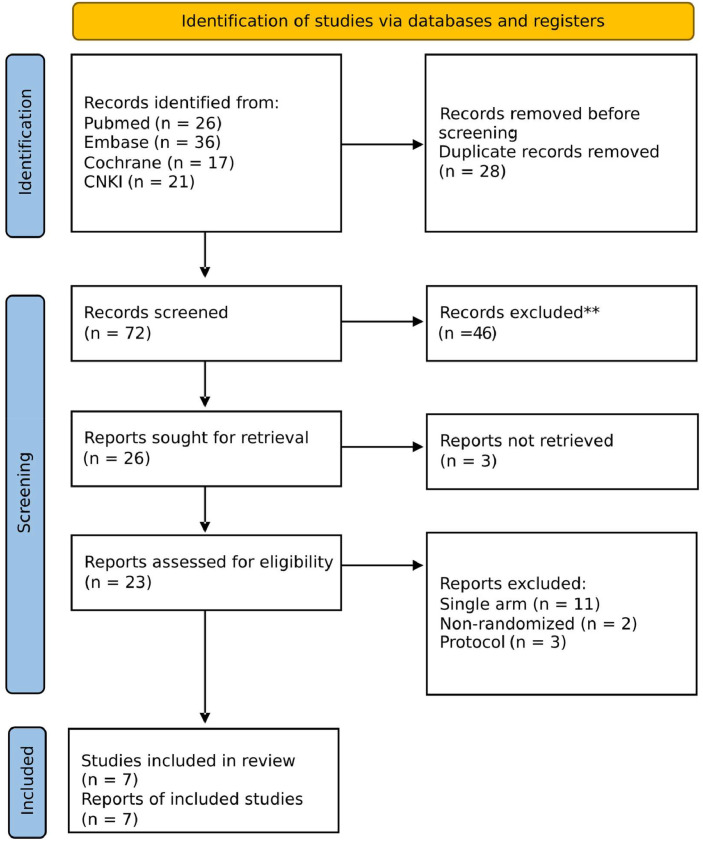
Flow diagram illustrating the study selection process according to the PRISMA guidelines.

As shown in Figure 1, the initial search identified 100 studies. After removing duplicates and ineligible studies, 24 remained and were fully reviewed based on the inclusion criteria. Of these, 7 RCTs^21^, ^22^, ^23^, ^24^, ^25^, ^26^, ^27^ were included, comprising 362 individuals with Herpes Zoster-related pain. Participants were equally divided between those receiving ESPB combined with clinical therapy and those receiving clinical therapy alone. Of the total, 192 had acute Herpes Zoster, while the remaining 170 patients had PHN. Medication therapy for those with acute infection included antiviral drugs (acyclovir or valacyclovir), either alone or in combination with pregabalin, gabapentin or methylcobalamin. For those with PHN, the primary medication used was pregabalin or gabapentin, either alone or combined with an anti-inflammatory. Further details on the study characteristics are reported in Table 1.

**Table 1:** Characteristics of included studies.

Study	Anesthetic Composition for ESPB and Application Frequency	Standard oral medicines	Patients ESPB / Control	Male (%) ESPB / Control	Phase of pain in study	Age^a^ (years) ESPB / Control	Baseline pain^b^ ESPB / Control	Follow-up, weeks	Country	Outcomes available
Patil^21^ 2024	20 mL Bup 0.25% + 8 mg Dex, applied only once	No specified	20 / 20	75 / 55	Acute	56.10 / 57.60	7.7 / 7.5	8	India	Incidence of PHN, pain score (2, 4 and 8 w), and rescue analgesic requirement.
Lin^22^ 2021	25 mL Rop. 0.4%, applied daily for 3 days	Valacyclovir + MeCbl	26 / 26	46.2 / 50	Acute	68.2 / 65.2	6 / 6.8	12	China	Incidence of PHN, pain score (1, 4, and 12 w), acetaminophen and tramadol consumption
Abdelwahab^23^ 2022	Epinephrine + 2.5 mL Bup 0.5% + 8 mg Dex, applied only once	Acyclovir + Pregabalin	30 / 30	40 / 43.3	Acute	59.47 / 61.3	7 / 7	24	Egypt	Incidence of PHN, pain score (1, 3, 4, 12, and 24 w), pregabalin and acetaminophen consumption
Ahmed^24^ 2022	20 mL Bup 0.25%, applied only once	Pregabalin + Acetaminophen	25 / 25	56 / 52	PHN	56.16 /54.36	7 / 7	12	Egypt	Pain score (each week until 12 weeks), pregabalin and acetaminophen consumption
El-Sayed^25^ 2021	20 mL Bup 0.25%, applied once: after 2 w, second application, if VAS > 6	Acyclovir + Pregabalin	20 / 20	N/A	Acute	N/A	8.8 / 9	12	Egypt	Incidence of PHN, pain score (2, 4, and 12 w), pregabalin and acetaminophen consumption
Xiang^26^ 2018	20 mL Rop 0.15% + 0.3 mL Beta + 0.5 g MeCbl Applied once; repeat at 2 w or 4 w if needed.	Gabapentin	30 / 30	N/A	PHN	71.2/71.5	7.59 / 7.54	10	China	Pain score (1, 4, 6, 8, and 10 w)
Cao^27^ 2019	20 mL Rop 0.5% + 20 mg Triamci + 0.5 mg MeCbl Applied weekly for 4 w	Pregabalin	30 / 30	46.67 / 50	PHN	65/65	7.3 / 7.4	8	China	Pain score (1, 2, 3, 4, 5, 6 and 8 w), pregabalin consumption

ESPB, Erector Spinae Plane Block; PHN, Postherpetic Neuralgia; Rop, Ropivacaine; Bup, Bupivacaine; Dex, Dexamethasone; Beta, Betamethasone; Triamci, Triamcinolone; MeCbl, Methylcobalamin; N/A, Not Available.

a Mean age;

b Pain using VAS (0‒10) and reporting mean.

### Pooled analysis of all studies and subanalysis

**Figure 2:**
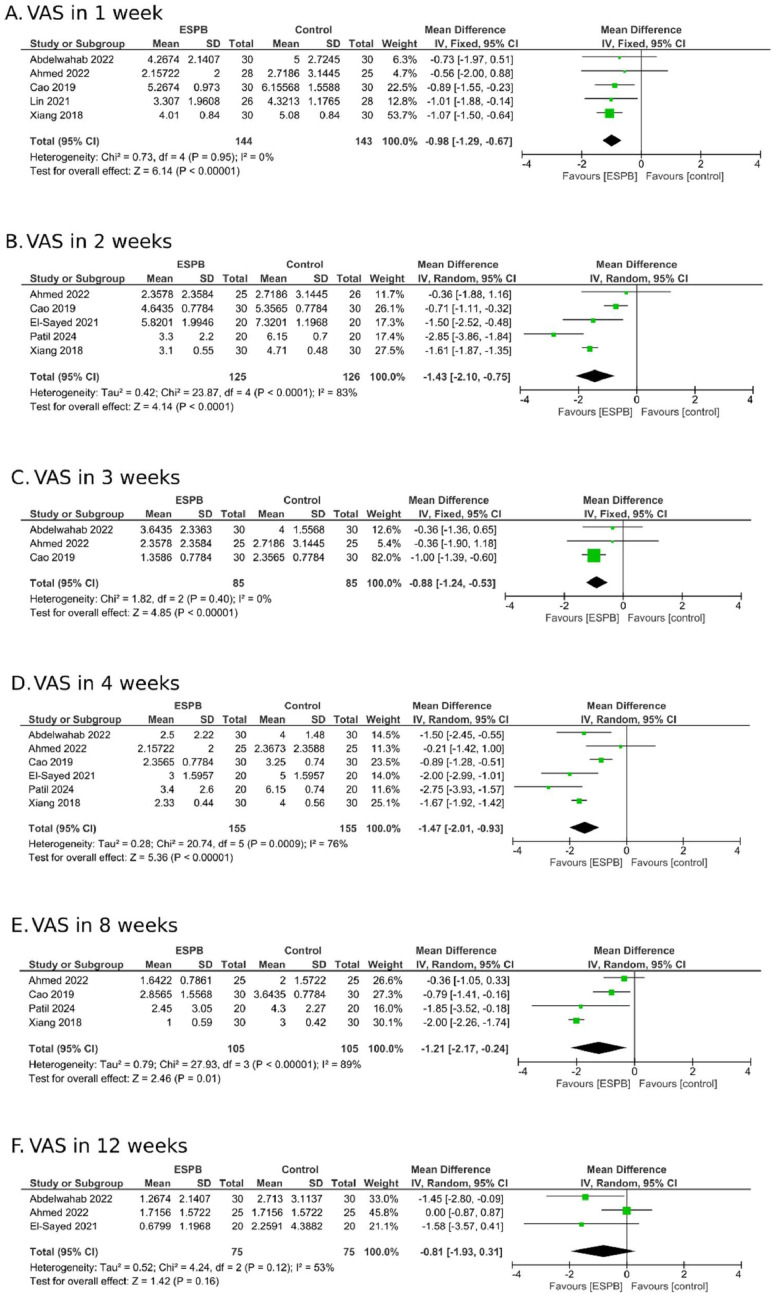
Analysis of ESPB therapy in reducing pain in patients with herpes zoster-related pain across several weeks of follow-up.

This meta-analysis of RCTs demonstrates that combining ESPB with clinical treatment is more effective than medication alone in controlling pain. One week after therapy, the MD in the VAS scale between the ESPB and control group was -0.98 (95% CI -1.29 to -0.67; I^2^ = 0%; Fig. 2A). Two weeks after, it was -1.43 (95% CI -2.10 to -0.75; I^2^ = 83%; Fig. 2B); three weeks after, it was -0.88 (95% CI -1.24 to -0.53; I^2^ = 0%; Fig. 2C); four weeks after, it was -1.47 (95% CI -2.01 to -0.93; I^2^ = 76%; Fig. 2D). At eight weeks, the MD was -1.21 (95% CI: -2.17 to -0.24; I^2^ = 89%; Fig. 2E). However, after this period, no statistical significance was observed. At 12 weeks, the effect size was -0.81 (95% CI -1.93 to 0.31; I^2^ = 53%; Fig. 2F).

**Figure 3:**
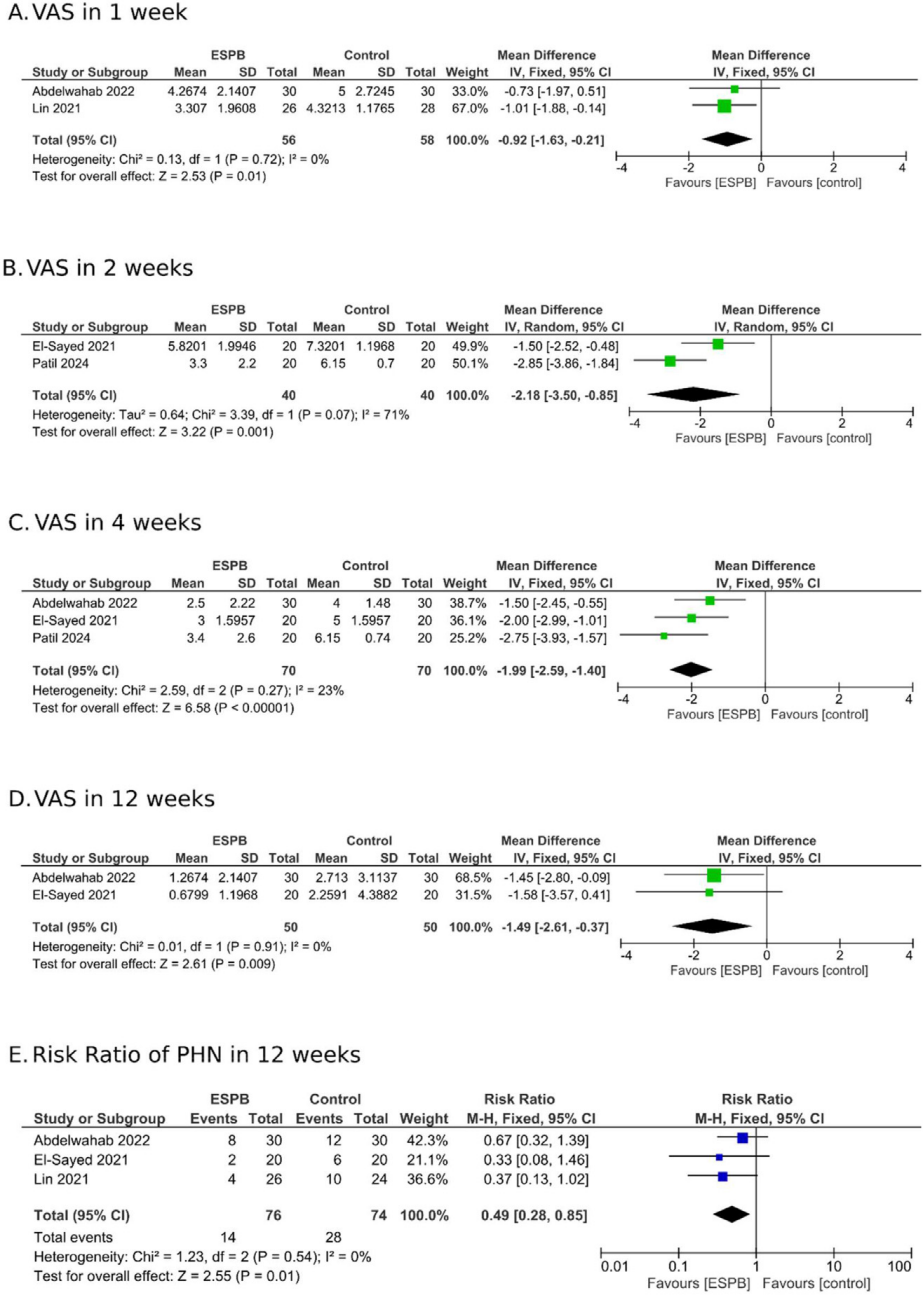
Subgroup analysis of ESPB therapy in patients in the acute phase of herpes zoster across several weeks of follow-up. (A–D) Analysis of pain reduction. (E) Risk ratio for the development of Postherpetic Neuralgia (PHN).

In the subanalysis of the acute phase of Herpes Zoster, four studies^21^, ^22^, ^23^^,^^25^ reported pain scale data. One week after the follow-up began, the MD between the groups was -0.92 (95% CI -1.63 to -0.21; I^2^ = 0%; Fig. 3A). Two weeks later, this value was -2.18 (95% CI -3.5 to -0.85; I^2^ = 71%; Fig. 3B). In four weeks, this value was -1.99 (95% CI -2.59 to -1.40; I^2^ = 23%; Fig. 3C), reinforcing the benefit of ESPB in pain management. Unlike the overall pooled result mentioned above, the subgroup analysis of patients with acute-phase herpes zoster showed pain score reduction even after 12 weeks, with a MD of -1.49 (95% CI -2.61 to -0.37; I^2^ = 0%; Fig. 3D). This finding is further supported by the combination of three studies^22^^,^^23^^,^^25^ that evaluated the number of patients that developed PHN at the third month of follow-up. The risk ratio of 0.49 (95% CI 0.28 to 0.85; I^2^ = 0%; Fig. 3E) indicates a significant reduction PHN in patients undergoing ESPB.

**Figure 4:**
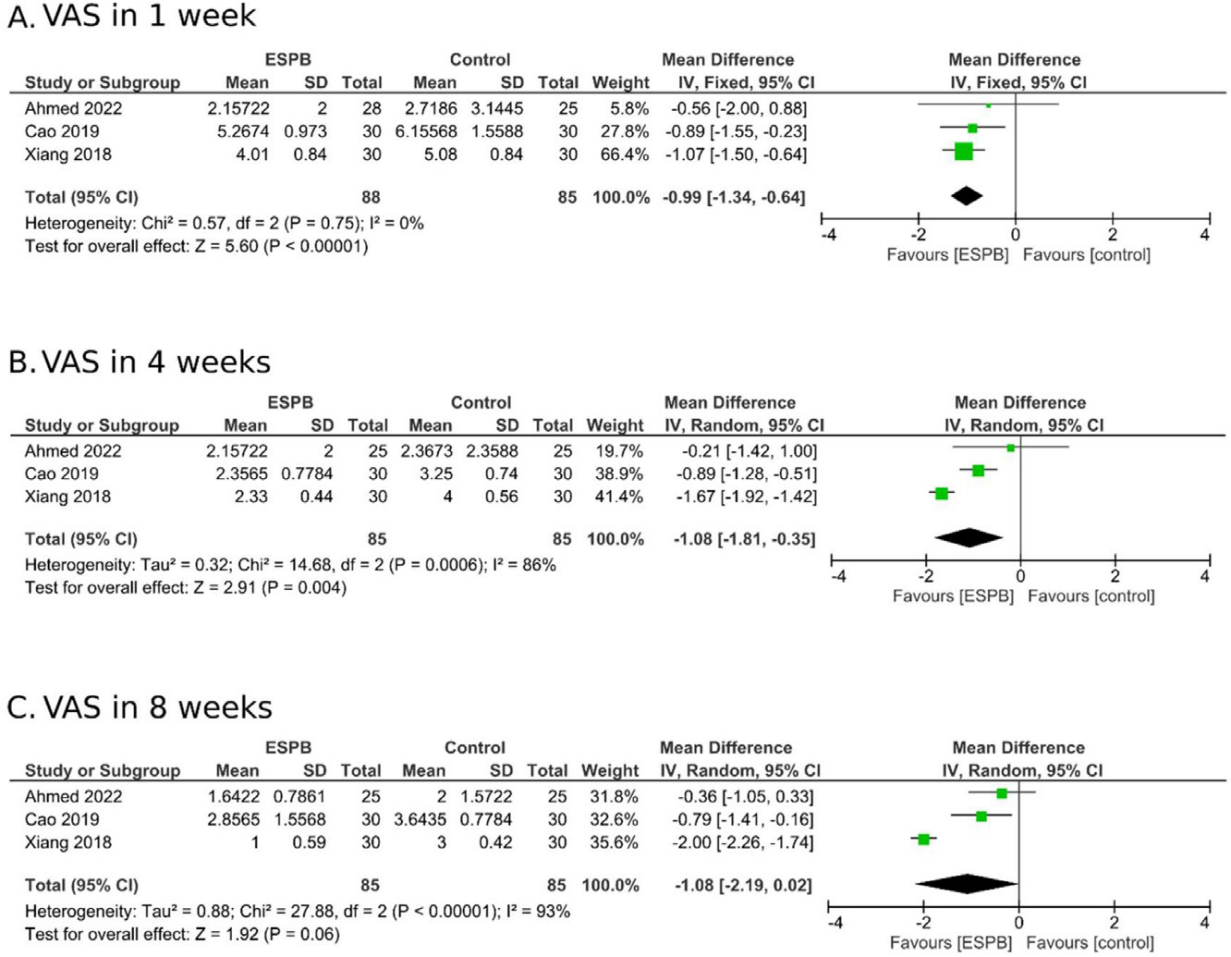
Subgroup analysis of ESPB therapy in patients with Postherpetic Neuralgia (PHN).

In the subgroup analysis of patients with PHN, the MD one week after ESPB was -0.99 (95% CI -1.34 to -0.64; I^2^ = 0%; Fig. 4A). At four weeks, the MD was -1.08 (95% CI -1.81 to -0.35; I^2^ = 86%; Fig. 4B). However, by eight weeks, the MD was -1.08 (95% CI -2.19 to 0.02; I^2^ = 93%; Fig. 4C), demonstrating no clear conclusions regarding its benefit at this time point. Beyond this period, only one study^24^ assessed pain scores in patients with PHN and demonstrated pain score reduction only up to 1 week.

**Figure 5:**
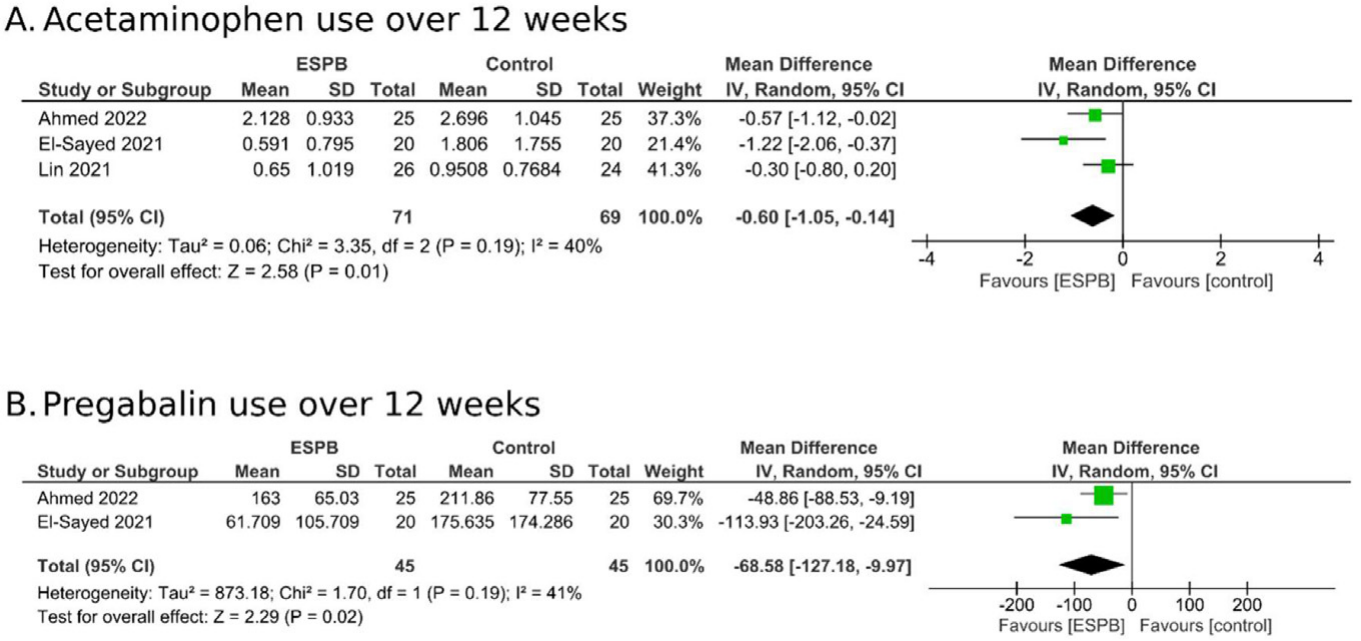
Mean Difference (MD) in the consumption of analgesics and neuroleptic medications.

Regarding analgesic medication, three studies^22^^,^^24^^,^^25^ reported the daily dose of acetaminophen over 12 weeks. Our meta-analysis revealed that patients undergoing ESPB required a lower amount of this analgesic, with an MD of -0.60 g per day (95% CI -1.05 to -0.14; I^2^ = 49%; Fig. 5A). Additionally, one study^23^ evaluated the total dose of acetaminophen used over 24 weeks of follow-up, showing that the ESPB group had a significantly lower total dose of this analgesic (78.53g vs. 153.83g, p = 0.024). Only one study^22^ evaluated opioids, demonstrating a significant difference in daily tramadol consumption over 12 weeks as well (36 mg vs. 245 mg, p = 0.001).

Additionally, two studies^24^^,^^25^ assessed the daily use of pregabalin over 12 weeks and, through analysis of the combined data, indicated that patients undergoing ESPB required a lower dose of this anticonvulsant, with a reduction of -68.58 mg per day (95% CI -127.18 to -9.97; I^2^ = 41%; Fig. 5B). Two other studies^23^^,^^27^ evaluated the total dose of pregabalin. In the study conducted by Cao^27^ patients in the intervention group required a significantly lower total amount of pregabalin over the 4-week evaluation period (1.8 g vs. 7.28 g; p < 0.005). In the study conducted by Abdelwahab,^23^ the same medication was also significantly lower in the intervention group over a 24-week period (24,550.8 mg vs. 35,575.0 mg, p = 0.041).

Regarding minor adverse effects such as dizziness, drowsiness, and nausea, three studies^24^^,^^26^^,^^27^ reported these complications, with the combined statistical analysis showing no significant difference between ESPB and placebo (RR = 0.71; 95% CI 0.36 to 1.38; I^2^ = 0%; Supplementary Fig. 1). Finally, no major complications, such as pneumothorax or bleeding, were reported in the studies.

### Quality assessment

Individual RCT appraisal is reported in Supplementary Figure 2. Two^22^^,^^24^ of the six RCTs included in this systematic review were assessed as having a low risk of bias. These trials were double-blinded and clearly described an adequate randomization process. Another three studies^21^^,^^23^^,^^25^ were rated as having some concerns, primarily because they did not provide sufficient information regarding the blinding of outcome assessors and patients, raising uncertainty about the potential for bias in this domain. The remaining trials^26^^,^^27^ were assessed as having a high risk of bias because, in addition to not mentioning the blinding of assessors and patients, they also did not provide a detailed explanation of how the randomization process was conducted, and they did not have a study protocol before the initiation of RCT registered in Clinical Trials.

## Discussion

Our article is the first systematic review and meta-analysis conducted to evaluate whether ESPB therapy can improve clinical symptoms in patients with herpes zoster-related pain. This approach highlights the potential of ESPB as an alternative management strategy, particularly for cases unresponsive to conventional pharmacological treatments. The key findings demonstrate that ESPB therapy resulted in substantial pain reduction, improved patient-reported outcomes, and significantly decreased the need for analgesics, notably pregabalin, acetaminophen, and tramadol. Additionally, no adverse effects were reported, reinforcing the safety profile of this intervention.

In the combined analysis of all included studies, significant heterogeneity was identified in pain outcomes, particularly at the second, fourth, and eighth weeks of clinical follow-up as evidenced in Figure 2. This variability can be attributed to differences in the studied populations, as the pooled analysis included patients in the acute phase of herpes zoster and those with PHN. In the acute phase, pain is primarily inflammatory, driven by varicella-zoster virus reactivation and local immune responses, resulting in peripheral sensitization.^28^^,^^29^ In contrast, postherpetic neuralgia is characterized by chronic neuropathic pain, stemming from nerve damage, demyelination, and central nervous system remodeling.^30^, ^31^, ^32^, ^33^ These distinct mechanisms likely influenced treatment efficacy and contributed to the observed heterogeneity, highlighting the need to consider disease stage in interpreting pooled outcomes.

Another factor that justifies the observed heterogeneity is the varying methods by which ESPB was performed in the intervention group across the included studies. For example, three studies^21^^,^^23^^,^^24^ analyzed a single injection of ESPB. On the other hand, other studies adopted repeated applications of ESPB, ranging from a second application in the fourth week, depending on the patient's pain level, to weekly applications during the first four weeks. This introduces significant variability in pain control and management across studies, increasing heterogeneity, particularly when analyzed over longer follow-up periods, especially after 4 weeks. Another reason for high heterogeneity is the composition of ESPB. Some studies utilized adjuvant medications alongside local anesthetics during ESPB. For instance, Patil et al.^21^ performed the block using bupivacaine combined with 8 mg of dexamethasone, while Xiang et al.^26^ employed ropivacaine with 0.3 mL of betamethasone. According to previous meta-analyses^34^^,^^35^ and randomized trials^35^ in other clinical contexts, perineural corticosteroids can prolong the duration of the block and enhance the efficacy of local anesthetics. Given the inflammatory nature of herpes zoster-associated pain, the use of corticosteroids in ESPB may further potentiate its effect. Therefore, another factor that could influence and contribute to the observed heterogeneity is the inclusion of studies^27^^,^^26^ that utilized perineural administration of methylcobalamin alongside the anesthetic, which may introduce variability in outcomes. The perineural use of methylcobalamin in combination with local anesthetics has been investigated in studies^36^^,^^37^ addressing herpes zoster-related pain, demonstrating significant pain relief. Methylcobalamin, the active form of vitamin B12, exhibits neuroprotective effects, facilitates neuronal regeneration, and reduces nerve hyperexcitability, thereby alleviating neuropathic pain.

To explore heterogeneity, ESPB was evaluated in the acute phase of the disease as well as in patients with PHN. In the acute stage, the results demonstrated greater homogeneity of outcomes and more prolonged pain control, lasting up to 12 weeks, as assessed by the combined analysis of studies in this phase. This can be attributed to patients in the acute phase who underwent the block experiencing a lower rate of pain chronification, defined as PHN over 12 weeks. In other words, fewer patients in the ESPB group reported persistent pain, leading to a reduction in pain scale scores. This finding is supported by the observed RR of 0.49, as depicted in Figure 2E, which corroborates this assertion. A brief review of the literature analyzing other types of nerve blocks reveals similar findings. A meta-analysis conducted by Kim et al.^7^ reported comparable results regarding the risk of PHN in patients in the acute phase who underwent paravertebral block, a technique that shares a similar mechanism of action with ESPB. In this study, an RR of 0.37 was observed, with a p-value of 0.01.

Regarding the subgroup of patients with PHN, only three studies^24^^,^^26^^,^^27^ were included, and heterogeneity remained high, primarily due to differences in block application frequency. Ahmed et al.^24^ performed a single application and observed pain scale improvements only during the first week of follow-up, with outcomes analyzed at 12 weeks. In contrast, Cao et al.^27^ administered weekly blocks during the first 4 weeks, and Xiang et al.^26^ applied the block at 2 or 4 weeks based on pain levels, resulting in benefits lasting up to 8 weeks. These variations significantly influenced the combined results, as shown in the forest plot. Notably, heterogeneity was zero at the 1 week follow-up, when all intervention groups received a single block. However, heterogeneity increased substantially at 4 and 8 weeks, likely due to additional applications in two studies.^26^^,^^27^ Despite these differences, the studies demonstrated pain relief, particularly during the first week of treatment. An interesting observation is that repeated applications resulted in prolonged pain control, as indicated by two studies,^27^^,^^26^ lasting up to 8 weeks of follow-up, even though the last application was administered in the fourth week. In other words, this approach provided an additional month of pain relief without any further interventions.

The ESPB significantly reduced the use of analgesic medications. The available data from the included studies primarily evaluated pregabalin, acetaminophen, and tramadol, highlighting a substantial reduction in analgesic dependence when ESPB was employed. This reduction in analgesic use was tracked in studies for up to 12 weeks. Notably, ESPB in neuropathic conditions, as presented in this review, underscores the need for further research. This is particularly important as avoiding the excessive use of systemic medications with potential adverse effects is critical in clinical practice, especially in elderly patients, often burdened with additional comorbidities and medication. Analgesics such as acetaminophen and tramadol can cause serious side effects when taken in excess.^38^ Excessive consumption of acetaminophen can be particularly associated with severe liver damage, including acute liver failure, as well as allergic reactions and, in rare cases, severe skin conditions like Stevens-Johnson syndrome.^39^^,^^40^ Opioids, on the other hand, are linked to both dependence and addiction, alongside notable side effects such as nausea and constipation.^41^

The ESPB, a recently developed therapy by Forero et al.,^42^ has been gaining traction and is based on the injection of anesthetic between the erector spinae muscle and the transverse process of the vertebra, guided by an ultrasound device.^43^ This technique differs from other types of blocks, such as epidural and paravertebral blocks, due to its ease of execution and relative safety. One feared complication common to any type of anesthetic block is the risk related to the systemic absorption of local anesthetics, which can result in respiratory depression and central nervous system effects. These risks can be mitigated by adhering to the maximum allowable dose of the anesthetic and employing simple measures, such as aspirating the needle before injecting. This review did not identify any major adverse effects, including pneumothorax and hematoma. Minor side effects, such as nausea and dizziness, were comparable between the intervention and control groups.

This study has several limitations that need to be highlighted. Firstly, significant heterogeneity was identified in the combined analysis, possibly reflecting disparities in how the intervention was performed across the studies. To address this issue, a subgroup analysis was conducted; however, due to the limited number of studies and, consequently, the small number of patients analyzed, drawing precise conclusions remains challenging. Another factor that contributed to the heterogeneity was the varying follow-up periods across the studies, with some evaluating the intervention only at 8 weeks while others extended it to a longer period. The frequency of clinical follow-ups also varied, with some studies reporting outcomes weekly, while others used more spaced intervals. Consequently, when the outcomes were combined, this generated a publication bias.

Another notable issue is the imprecision of the statistical calculations. Some studies required the conversion of medians and interquartile ranges into means and standard deviations. Although this was done using the Cochrane-recommended method, such conversions often distort results due to skewed data. Therefore, the findings presented in this study should be interpreted with caution.

The fact that all studies were conducted in only two countries, China and India, introduces a potential bias that limits the generalizability and applicability of the findings. Indeed, research restricted to specific geographic regions may introduce publication biases related to the healthcare systems, demographic characteristics, and genetic factors of the analyzed populations. In terms of study quality, it is important to highlight that two out of the seven studies included were categorized as having a high risk of bias. This stems, in part, from the inclusion of peer-reviewed trials and studies present in the gray literature. While gray literature can provide valuable insights and broaden the scope of the review, its inclusion introduces severe limitations. Specifically, gray literature often contains studies with inconsistent methodological rigor and may not meet the highest standards of quality.

Finally, the outcomes related to analgesic consumption were also notably limited. This is because the studies evaluated only the use of pregabalin, acetaminophen, and tramadol, without considering other medications such as gabapentin and other opioids.

## Conclusion

In this review, ESPB appears to be effective in reducing pain and the use of analgesic medications in patients with herpes zoster-related pain. The potential benefits seem to be more prolonged during the acute phase of the disease. However, further studies with standardized ESPB application protocols are necessary to validate and better understand these findings.

## Conflicts of interest

The authors declare no conflicts of interest.

## Appendix Supplementary materials

Image, application 1

## Footnotes

Supplementary material associated with this article can be found in the online version at doi:10.1016/j.bjane.2025.844598.

## References

[1] Lim DZJ, Tey HL, Salada BMA (2024). Herpes Zoster and Post-Herpetic Neuralgia-Diagnosis, Treatment, and Vaccination Strategies. Pathog. Basel Switz..

[2] John A.R., Canaday D.H. (2017). Herpes Zoster in the Older Adult. Infect. Dis. Clin. North Am..

[3] Marra F, Parhar K, Huang B, Vadlamudi N. (2020). Risk Factors for Herpes Zoster Infection: A Meta-Analysis. Open Forum Infect. Dis..

[4] Zhou H, Wang Z, Jin H, Chen X, Lei L (2021). A systematic review and meta-analysis of independent risk factors for postherpetic neuralgia. Ann Palliat Med.

[5] Wang Q, Yang L, Li L, Liu C, Jin H, Lin L (2023).

[6] Katz J, Cooper EM, Walther RR, Sweeney EW, Dworkin RH. (2024). Acute pain in herpes zoster and its impact on health-related quality of life. Clin Infect Dis.

[7] Kim HJ, Ahn HS, Lee JY (2017). Effects of applying nerve blocks to prevent postherpetic neuralgia in patients with acute herpes zoster: a systematic review and meta-analysis. Korean J Pain.

[8] Kim J, Kim MK, Choi GJ, Shin HY, Kim BG, Kang H. (2021). Pharmacological and non-pharmacological strategies for preventing postherpetic neuralgia: a systematic review and network meta-analysis. Korean J Pain.

[9] Wen B, Wang Y, Zhang C, Xu W, Fu Z. (2020). Efficacy of different interventions for the treatment of postherpetic neuralgia: a Bayesian network meta-analysis. J Int Med Res.

[10] Slinchenkova K, Lee K, Choudhury S, Sundarapandiyan D, Gritsenko K. (2023). A Review of the Paravertebral Block: Benefits and Complications. Curr Pain Headache Rep.

[11] Surdhar I, Jelic T. (2022). The erector spinae plane block for acute pain management in emergency department patients with rib fractures. CJEM.

[12] Koo C-H, Lee H-T, Na H-S, Ryu J-H, Shin H-J. (2022). Efficacy of Erector Spinae Plane Block for Analgesia in Thoracic Surgery: A Systematic Review and Meta-Analysis. J Cardiothorac Vasc Anesth.

[13] Moher D, Liberati A, Tetzlaff J, Altman DG (2009). PRISMA Group. Preferred reporting items for systematic reviews and meta-analyses: the PRISMA statement. BMJ.

[14] Rosas S, Paço M, Lemos C, Pinho T. (2017). Comparison between the Visual Analog Scale and the Numerical Rating Scale in the perception of esthetics and pain. Int Orthod.

[15] Luo D, Wan X, Liu J, Tong T. (2018). Optimally estimating the sample mean from the sample size, median, mid-range, and/or mid-quartile range. Stat Methods Med Res.

[16] Wan X, Wang W, Liu J, Tong T. (2014). Estimating the sample mean and standard deviation from the sample size, median, range and/or interquartile range. BMC Med Res Methodol.

[17] Mood AM, Graybill FA, Boes DC. (1974).

[18] Snedecor GW, Cochran WG. (1989). Statistical Methods.

[19] Sterne JAC, Savović J, Page MJ (2019). RoB 2: a revised tool for assessing risk of bias in randomised trials. BMJ.

[20] McGuinness LA, Higgins JPT. (2021). Risk-of-bias VISualization (robvis): An R package and Shiny web app for visualizing risk-of-bias assessments. Res Synth Methods.

[21] Patil A, Vyshnavi S, Raja T, Shastry V, Thammaiah SH, Archana KN. (2024). A Randomized clinical trial comparing the efficacy of ultrasound-guided erector spinae block and paravertebral block in preventing postherpetic neuralgia in patients with zoster-associated pain. J. Anaesthesiol. Clin. Pharmacol..

[22] Lin Z-M, Wang H-F, Zhang F, Ma J-H, Yan N, Liu X-F. (2021). The Effect of Erector Spinae Plane Blockade on Prevention of Postherpetic Neuralgia in Elderly Patients: A Randomized Double-blind Placebo-controlled Trial. Pain Physician.

[23] Abdelwahab EH, Hodeib AA, Marof HM, Fattooh NH, Afandy ME. (2022). Ultrasound-Guided Erector Spinae Block Versus Ultrasound-Guided Thoracic Paravertebral Block for Pain Relief in Patients with Acute Thoracic Herpes Zoster: A Randomized Controlled Trial. Pain Physician.

[24] Ahmed SA, Magdy AA, Abdullah MA, Albadry AA. (2022). The Effect of Erector Spinae Plane Block With and Without Addition of Magnesium on Relief of Pain from Post-herpetic Neuralgia. Pain Physician.

[25] El-Sayed MA, Zanaty O, Shafshak W, Abdelmaksoud R, Gamal Eldine H. (2021). Role of ultrasound guided erector spinae plane block in management of acute herpes zoster pain and incidence of post-herpetic neuralgia. Egypt J Anaesth.

[26] Xiang Y, Liu F, Liu Y, Sun W (2018). Ultrasound-guided erector spinae plane block for the treatment of postherpetic neuralgia. Chinese Journal of Pain Medicine.

[27] Cao Y, Yue K, Zhang JX, Lin XW. (2019). Ultrasound-guided erector spinae plane block combined with pregabalin for post-herpetic neuralgia. Zhonghua Yi Xue Za Zhi.

[28] Patil A, Goldust M, Wollina U. (2022). Herpes zoster: A Review of Clinical Manifestations and Management. Viruses.

[29] Oaklander AL. (2018). Mechanisms of pain and itch caused by herpes zoster (shingles). J. Pain..

[30] Tang Y, Ren C, Wang M (2021). Altered gray matter volume and functional connectivity in patients with herpes zoster and postherpetic neuralgia. Brain Res.

[31] Liu J, Gu L, Huang Q (2019). Altered gray matter volume in patients with herpes zoster and postherpetic neuralgia. J Pain Res.

[32] Jiang X, Kuang H, Lv H (2023). Aberrant functional and causal connectivity of the amygdala in herpes zoster and post-herpetic neuralgia patients. Br J Radiol.

[33] Devor M. (2018). Rethinking the causes of pain in herpes zoster and postherpetic neuralgia: the ectopic pacemaker hypothesis. Pain Rep.

[34] De Oliveira GS, Castro Alves LJ, Nader A, Kendall MC, Rahangdale R, McCarthy RJ (2014). Perineural dexamethasone to improve postoperative analgesia with peripheral nerve blocks: a meta-analysis of randomized controlled trials. Pain Res Treat.

[35] Kirkham KR, Jacot-Guillarmod A, Albrecht E (2018). Optimal Dose of Perineural Dexamethasone to Prolong Analgesia After Brachial Plexus Blockade: A Systematic Review and Meta-analysis. Anesth Analg.

[36] Sawangjit R, Thongphui S, Chaichompu W, Phumart P. (2020). Efficacy and Safety of Mecobalamin on Peripheral Neuropathy: A Systematic Review and Meta-Analysis of Randomized Controlled Trials. J Altern Complement Med N Y N.

[37] Xu G, Zhou GS, Tang WZ (2020). Local Administration of Methylcobalamin for Subacute Ophthalmic Herpetic Neuralgia: A Randomized, Phase III Clinical Trial. Pain Pract.

[38] Chiew AL, Gluud C, Brok J, Buckley NA. (2018). Interventions for paracetamol (acetaminophen) overdose. Cochrane Database Syst Rev.

[39] Larson AM. (2007). Acetaminophen hepatotoxicity. Clin. Liver Dis..

[40] Khawaja A, Shahab A, Hussain SA. (2012). Acetaminophen induced Steven Johnson syndrome-toxic epidermal necrolysis overlap. J Pak Med Assoc.

[41] Coussens NP, Sittampalam GS, Jonson SG (2019). The Opioid Crisis and the Future of Addiction and Pain Therapeutics. J Pharmacol Exp Ther.

[42] Forero M, Adhikary SD, Lopez H, Tsui C, Chin KJ. (2016). The erector spinae plane block a novel analgesic technique in thoracic neuropathic pain. Reg Anesth Pain Med.

[43] De Cassai A, Geraldini F, Freo U (2023). Erector Spinae Plane Block and Chronic Pain: An Updated Review and Possible Future Directions. Biology.

