# Supplementary material for: Efficacy of erector spinae plane block in pain management for patients with herpes zoster: a systematic review and meta-analysis
Source: Braz J Anesthesiol. 2025 Feb 18;75(2):844598. doi: 10.1016/j.bjane.2025.844598 (PMC11930083; doi:10.1016/j.bjane.2025.844598)

**BJAN-D-24-00522_Supplementary Material**

**Supplementary Table 1** Detailed search strategy used for each database included in the systematic review.

| **PUBMED:** (“erector spinae plane block” OR “erector spinae block”) AND ("herpes zoster"[MH] OR "herpes zoster" OR “herpes-zoster” OR "human herpesvirus 3" OR "varicella" OR "chickenpox" OR "varicellae" OR postherpetic) |
| --- |
| **Embase:** (“erector spinae plane block” OR “erector spinae block”) AND ("herpes zoster" OR herpes-zoster OR "human herpesvirus 3" OR "varicella" OR "chickenpox" OR "varicellae" OR postherpetic) |
| **Cochrane:** (“erector spinae plane block” OR “erector spinae block”) AND ("herpes zoster" OR herpes-zoster OR "human herpesvirus 3" OR "varicella" OR "chickenpox" OR "varicellae" OR postherpetic) |
| **CNKI:** 竖脊肌平面阻滞 与 (带状疱疹 或 带状疱疹神经痛) |

**Supplementary Figure 1** Risk ratio of minor adverse events between ESPB and control groups.


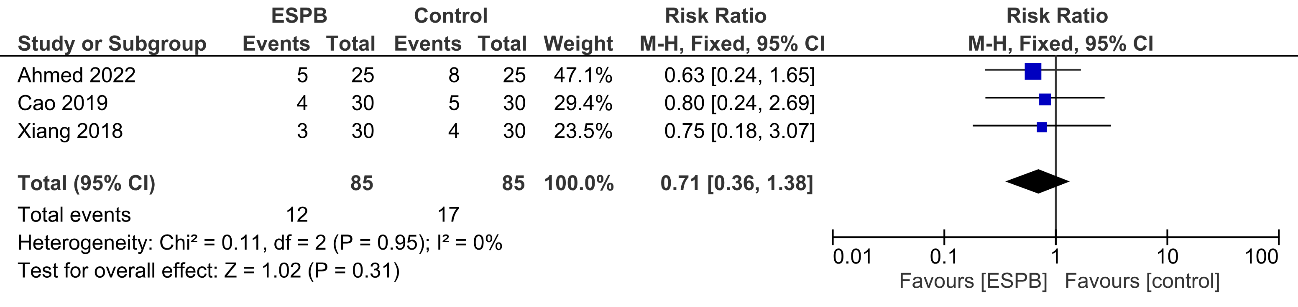


**Supplementary Figure 2** Risk of bias of included studies.


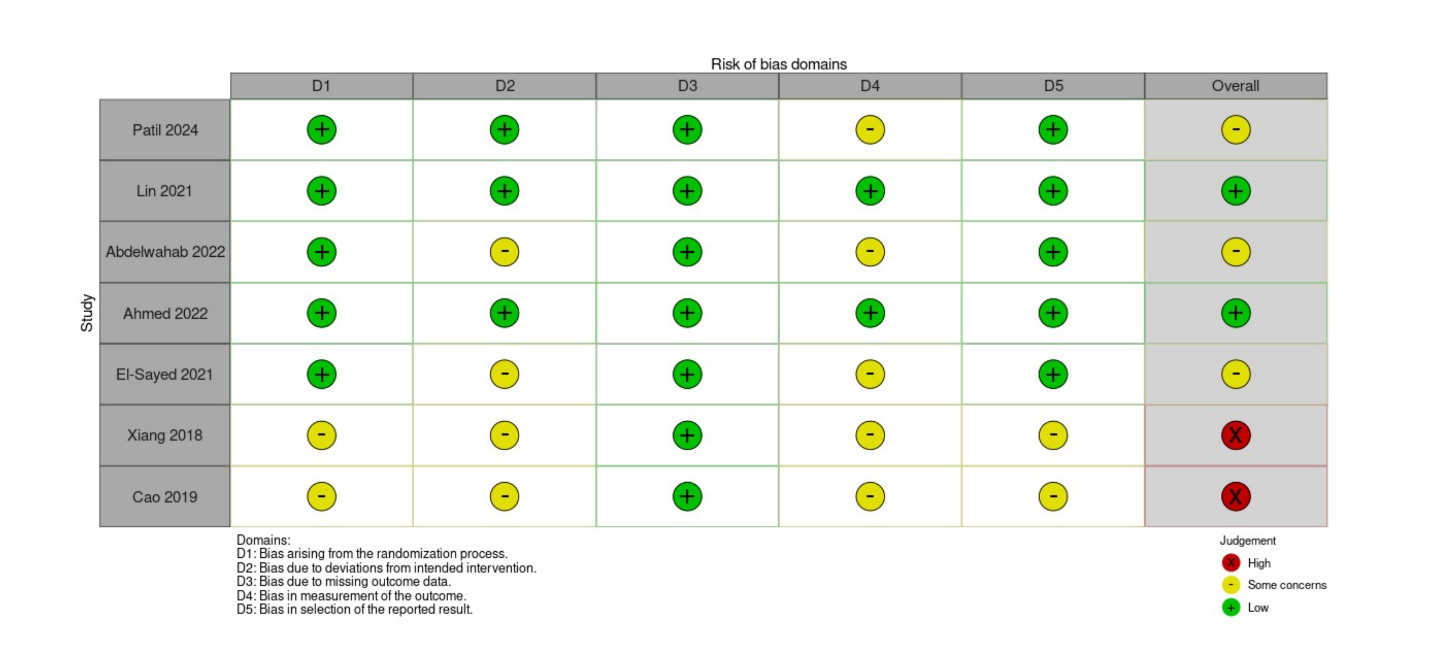

Supplement: Supplementary file 1 [file mmc1.docx]
